# O‐GlcNAcylation Stimulation Modulates Na+/K+ ATPase and Improves Outcomes in Hemorrhagic Shock

**DOI:** 10.1002/mco2.71056

**Published:** 2026-10-11

**Authors:** Thomas Dupas, Amandine Vergnaud, Virginie Aillerie, Martin Bouaud, Angélique Erraud, Thomas Pelé, Antoine Persello, Barbara Ribeiro de Oliveira, Jérôme Montnach, Aurore Girardeau, Jules Lecomte, Nolwenn Tessier, Mathilde Guyon, Angélique Blangy‐Letheule, Matthieu Rivière, Jacques Lebreton, Arnaud Tessier, Edith Bigot‐Corbel, Aurélia A. Leroux, Michel De Waard, Nicolas J. Prat, Bertrand Rozec, Manon Denis, Benjamin Lauzier

**Affiliations:** ^1^ Nantes Université, CHU Nantes, CNRS, INSERM, L'institut Du Thorax Nantes France; ^2^ Nantes Université, CNRS, CEISAM, UMR 6230, 2 Rue De La Houssinière Nantes France; ^3^ Nantes Université, CHU de Nantes, Department of Biochemistry Nantes France; ^4^ Oniris Nantes France; ^5^ Institut De Recherche Biomédicale Des Armées (IRBA) Bretigny‐sur‐Orge France

**Keywords:** hemorrhagic shock, Na^+^/K^+^ ATPase, O‐GlcNAcylation, therapeutic strategy

## Abstract

Hemorrhagic shock (HS), caused by blood loss leading to inadequate organ perfusion, remains a major cause of death worldwide, with 1.9 million fatalities annually. It triggers severe disturbances in cardiovascular, neurohormonal, and metabolic homeostasis with ionic imbalance notably due to Na^+^/K^+^‐ATPase dysfunction. O‐GlcNAcylation, a post‐translational modification essential for cellular protection under stress, has emerged as a promising therapeutic target in ischemia‐reperfusion and septic shock.

This study aimed to determine the effects of stimulating O‐GlcNAcylation during HS and to clarify the related molecular mechanisms. Adult male rats were subjected to controlled HS and received either NButGT (2 injections of 10 mg/kg) to enhance O‐GlcNAcylation or saline. Hemodynamic parameters and plasmatic markers of organ injury were assessed after 24 h. O‐GlcNAcylation levels, protein expression, and Na^+^/K^+^‐ATPase O‐GlcNAcylation were analyzed via Western blot and immunofluorescence, while Na^+^/K^+^‐ATPase activity was measured by colorimetry. NButGT increased cardiac and renal O‐GlcNAcylation levels, improved hemodynamic and reduced organ damage. HS disrupts electrolyte homeostasis with decreased bicarbonate and sodium levels and elevated potassium concentration. NButGT administration restored ionic balance and renal Na^+^/K^+^‐ATPase expression. Treatment with NButGT elevated Na^+^/K^+^‐ATPase activity. These results demonstrate that acute O‐GlcNAcylation stimulation supports physiological recovery in HS by promoting ionic homeostasis and Na^+^/K^+^‐ATPase function.

## Graphical abstract

Acute stimulation of O‐GlcNAcylation via NButGT injections in rats subjected to hemorrhagic shock improves hemodynamic parameters, reduces organ dysfunction, and restores ionic imbalance. These beneficial effects are notably mediated through modulation of the Na^+^/K^+^‐ATPase pump, highlighting O‐GlcNAcylation as a promising therapeutic target in hemorrhagic shock.

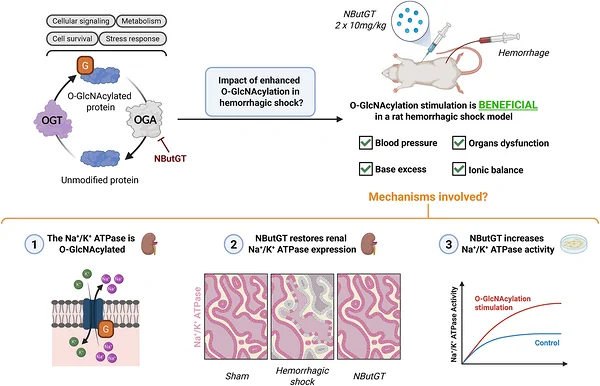

## 1 Introduction

Shock is defined as an acute circulatory failure leading to an organ hypoperfusion and an inability to meet cells' requirements for oxygen and nutrients [1, 2]. Hemorrhagic shock is characterized by a severe blood loss from various etiologies such as traumatic situation, rupture of an aneurysm, perioperative, gastrointestinal or delivery hemorrhage [3]. Each year, worldwide, 1.9 million deaths and 85 million years of life lost are imputed to hemorrhage and its consequences [3]. In addition to being one of the most important preventable causes of death in the world, hemorrhagic shock is associated with a quick mortality with a median time to hemorrhagic deaths of 2.0–2.6 h and 85% of deaths which occur within 6 h from the hospital admission [4, 5].

Mechanistically, the tissue hypoperfusion triggers a compensatory sympathetic response, increasing heart rate, cardiac contractility, and peripheral vasoconstriction. This vascular response redistributes blood flow toward vital organs (heart, brain) at the expense of others (kidneys, lungs, liver, bowel) [2]. As hemorrhagic shock progresses, reduced cardiac output impairs oxygen delivery, culminating in multiple organ dysfunction and death. Cellular oxygen deficiency shifts metabolism from aerobic to anaerobic, depleting energy stores and accumulating lactate, inorganic phosphates, and reactive oxygen species [6]. The complex pathophysiology of the hemorrhagic shock combines endothelial alteration, pro‐ and anti‐inflammatory, neurohormonal, metabolic and cardiovascular modifications to the lethal triad (acidosis, hypothermia and coagulopathy) [7]. Moreover, hemorrhagic shock is associated with an alteration of the ionic balance, notably due to a disruption of the Na^+^/K^+^ ATPase (NKA) activity which is essential for establishing and maintaining the electrochemical membrane potential and all related biological processes [8, 9]. The management of hemorrhagic shock remains challenging and although transfusion addresses the underlying cause, current treatments are largely symptomatic and often associated with adverse effects [5, 10, 11, 12, 13, 14]. In this sense, a new therapeutic strategy, based on a systemic approach would possibly limit tissue damage and could be beneficial in hemorrhagic shock.

O‐GlcNAcylation (O‐GlcNAc) is a post‐translational modification targeting the hydroxyl groups of serine, threonine and tyrosine residues of proteins, often modulating or competing with phosphorylation [15, 16, 17, 18, 19]. The GlcNAc moiety is added or hydrolyzed on proteins by O‐GlcNAc transferase (OGT) and O‐GlcNAcase (OGA), respectively. O‐GlcNAcylated proteins, localized in the cytosol, nucleus, mitochondria, and even extracellular compartments, are implicated in a wide range of biological processes, with an estimated 9000 modified proteins identified in human tissues as of 2025 [20, 21, 22].

Acute stimulation of O‐GlcNAc levels has been reported as protective in ischemia–reperfusion as well as in septic and hemorrhagic shock with a reduction of the inflammation and an increase in the survival rate [23, 24, 25, 26, 27, 28, 29, 30, 31, 32, 33, 34, 35, 36, 37, 38, 39, 40]. However, most studies have described the beneficial effects of the O‐GlcNAcylation over a very short period (2 h) after the hemorrhagic shock [30, 32, 33]. Only one study demonstrated the beneficial effects of the O‐GlcNAcylation 24 h post‐hemorrhage without reporting a difference in cardiac O‐GlcNAcylation levels, nor assessing hemodynamic parameters [29]. Moreover, the precise molecular mechanisms by which O‐GlcNAcylation stimulation exerts its beneficial effects are still poorly characterized. Given these shortcomings, our study aims to (1) evaluate the impact of O‐GlcNAcylation stimulation 24 h after hemorrhagic shock and (2) identify the molecular mechanisms associated with beneficial effects.

## 2 Results

### 2.1 NButGT Treatment Boosts Tissue O‐GlcNAcylation Levels

We first evaluated the impact of hemorrhagic shock and NButGT treatment on tissue O‐GlcNAcylation levels. Cardiac O‐GlcNAcylation levels remain unchanged following hemorrhagic shock (Sham: 1.00 ± 0.14; HS: 1.07 ± 0.20). Treatment with NButGT, an O‐GlcNAcase (OGA) inhibitor, significantly increased cardiac O‐GlcNAcylation levels (NButGT: 2.42 ± 0.51; *p* < 0.001) (Figure 1A). A similar pattern was observed in the kidney, where hemorrhagic shock did not impact O‐GlcNAcylation levels, but NButGT administration induced a significant increase (Sham: 1.00 ± 0.29; HS: 1.08 ± 0.20; NButGT: 1.54 ± 0.57; *p* < 0.05) (Figure 1B). These results demonstrate that NButGT efficiently increased tissue O‐GlcNAcylation levels.

**FIGURE 1:**
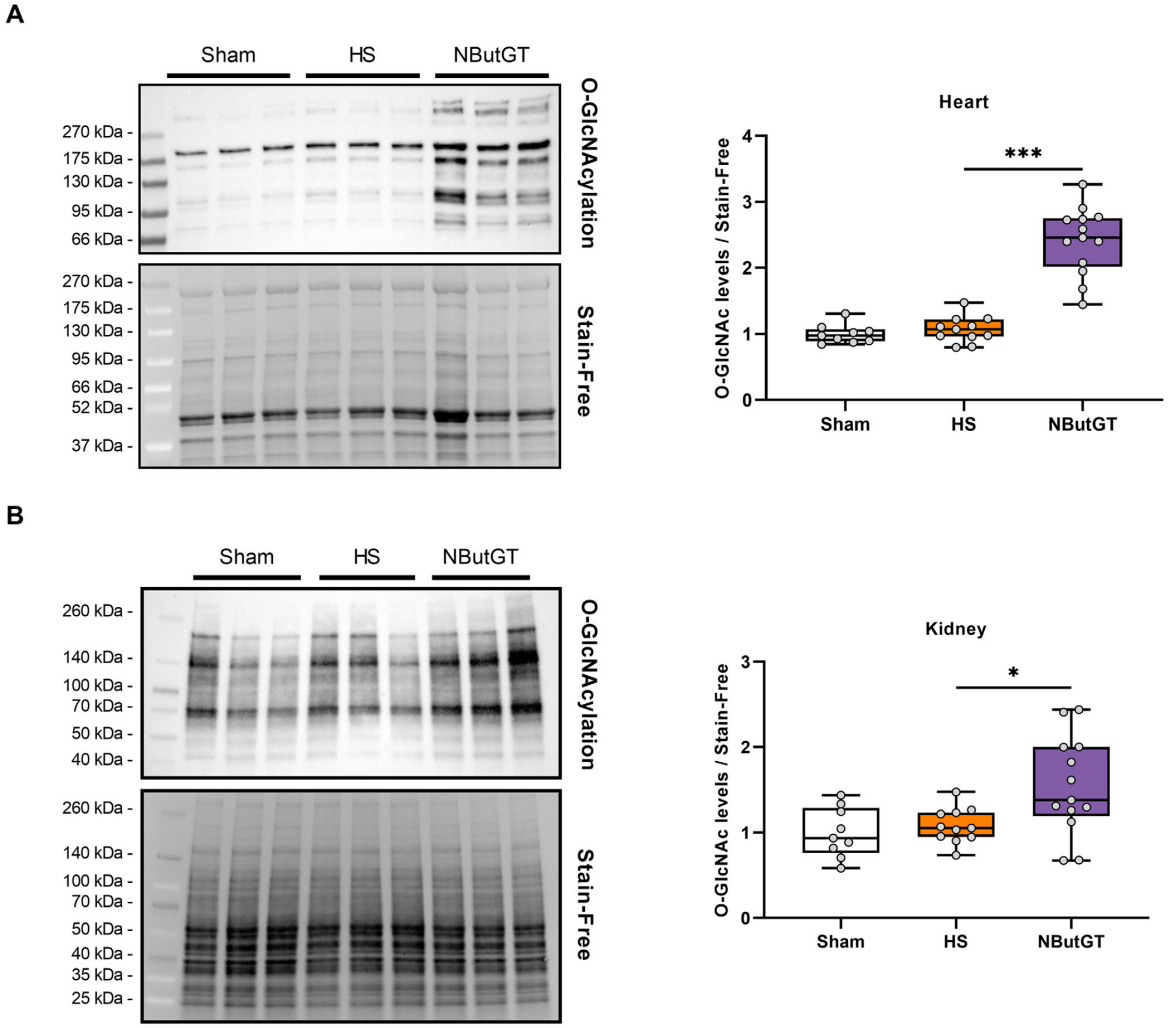
NButGT increases both cardiac and renal O‐GlcNAc levels in hemorrhagic shock. Evaluation by Western blot of (A) cardiac and (B) renal O‐GlcNAcylation levels 24 h after the hemorrhagic shock. The sham group underwent surgery only. Hemorrhagic shock group underwent hemorrhagic shock protocol. NButGT group underwent hemorrhagic shock with dual injection of NButGT at 10 mg/kg. Quantification of Western blot was related to Stain‐Free. Statistical analyses were assessed by Kruskal–Wallis test (A and B) HS: hemorrhagic shock. *: *p* < 0.05; ***: *p* < 0.001. *n* = 9–13.

### 2.2 Hemorrhagic Shock Increases Cardiac Expression of both GFAT1 and GFAT2

After demonstrating the efficacy of our pharmacological approach on O‐GlcNAcylation levels, we next examined whether the expression of key O‐GlcNAc enzymes was impacted. Cardiac OGT expression was not affected by hemorrhagic shock but was decreased in the NButGT‐treated group (Sham: 1.00 ± 0.15; HS: 0.89 ± 0.22; NButGT: 0.74 ± 0.13; *p* < 0.05) (Figure 2A). Cardiac OGA expression (quantified band indicated by an asterisk) was unmodified between the different studied groups (Sham: 1.00 ± 0.16; HS: 0.95 ± 0.16; NButGT: 0.99 ± 0.23) (Figure 2B). The expression of GFAT isoforms, the rate‐limiting enzymes of the hexosamine biosynthesis pathway, which generates the sugar donor used by OGT for protein O‐GlcNAcylation, showed a trend toward increased levels for GFAT1 and was increased for GFAT2 following hemorrhagic shock, while remained unchanged with NButGT treatment (GFAT1: Sham: 1.00 ± 0.18; HS: 1.56 ± 0.68; NButGT: 1.50 ± 0.43; GFAT2: Sham: 1.00 ± 0.34; HS: 2.40 ± 1.30; NButGT: 2.10 ± 0.80; *p* < 0.01) (Figure 2C,D). These results indicate that NButGT‐mediated OGA inhibition does not alter OGA expression, whereas GFAT1 and GFAT2 levels are specifically upregulated following hemorrhagic shock.

**FIGURE 2:**
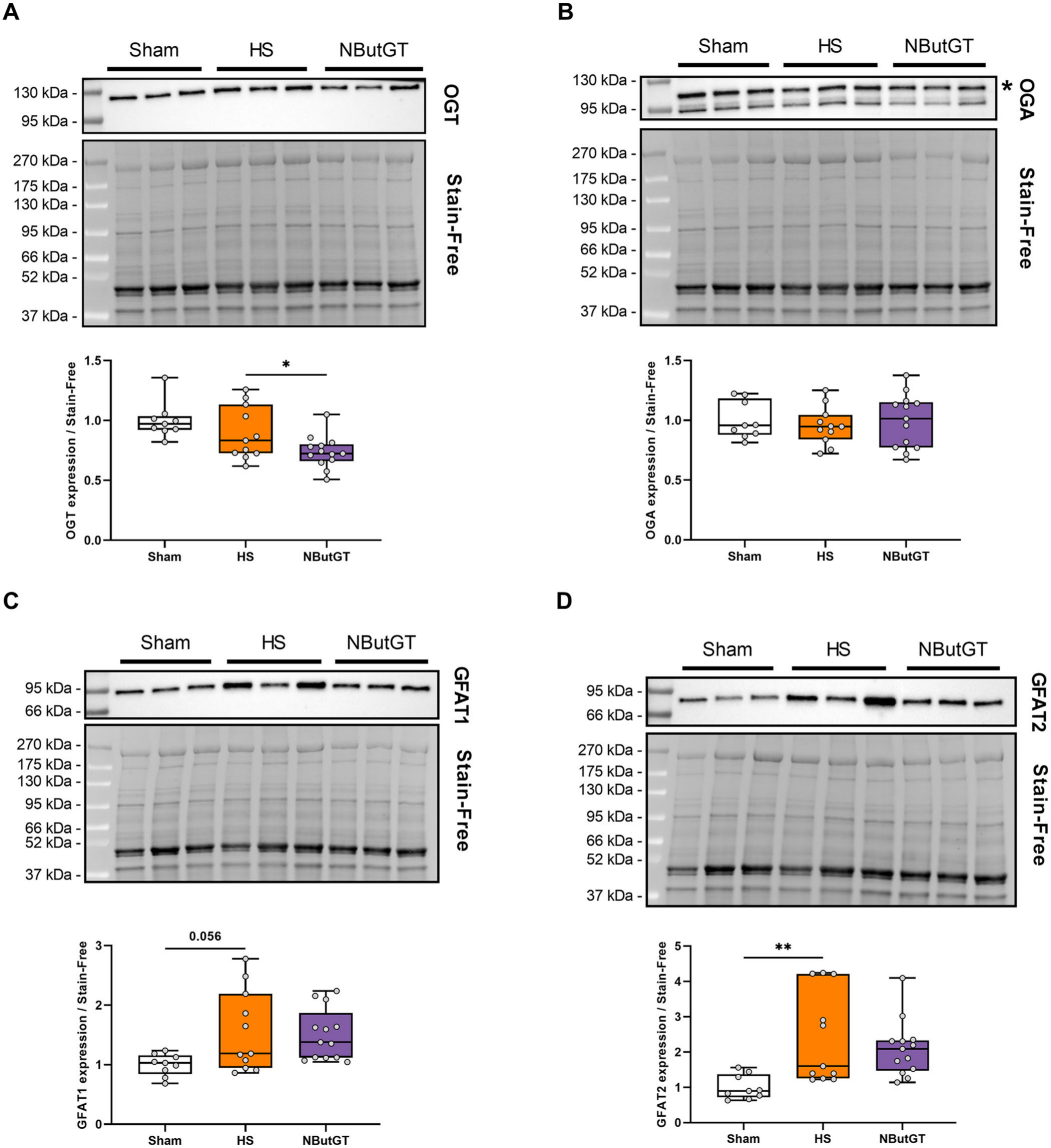
O‐GlcNAc associated enzymes are modulated with hemorrhagic shock. Evaluation by Western blot of cardiac expression of (A) OGT, (B) OGA, (C) GFAT1, and (D) GFAT2 24 h after the hemorrhagic shock. The sham group underwent surgery only. Hemorrhagic shock group underwent hemorrhagic shock protocol. NButGT group underwent hemorrhagic shock with dual injection of NButGT at 10 mg/kg. Quantification of Western blot were related to Stain‐Free. Statistical analyses were assessed by one‐way ANOVA (A and B) or Kruskal–Wallis test (C and D). HS: hemorrhagic shock. *: *p* < 0.05; **: *p* < 0.01. *n* = 9–13.

### 2.3 O‐GlcNAcylation Stimulation is Beneficial in Hemorrhagic Shock

Drop in blood pressure is a major hallmark of hemorrhagic shock. To determine the therapeutic potential of NButGT in this context, hemodynamic parameters were assessed 24 h after hemorrhagic shock. The basal mean arterial pressure was similar between different groups (Sham: 89 ± 5; HS: 89 ± 5; NButGT: 88 ± 6; mmHg) (Figure 3A). Twenty‐four hours after shock induction, mean arterial pressure was decreased in the hemorrhagic shock compared to the sham group (Sham: 97 ± 7; HS: 77 ± 13; mmHg; p<0.001) (Figure 3A). The NButGT treatment improved the mean arterial pressure (NButGT: 84 ± 7; mmHg; *p* < 0.05) mainly through an increase in the diastolic blood pressure (Sham: 79 ± 6; HS: 56 ± 10; NButGT: 63 ± 6; mmHg; *p* < 0.05) (Figure 3A,B). There were no significant differences in systolic blood pressure or heart rate among the groups (Figure 3C,D). The modified shock index, a predictor of mortality calculated by the ratio between heart rate and mean arterial pressure, was increased in the hemorrhagic shock group and was not significantly modified with NButGT treatment (Sham: 4.19 ± 0.54; HS: 5.42 ± 1.08; NButGT: 4.88 ± 0.44; *p* < 0.001) (Figure 3E). Together, these findings underscore that NButGT treatment helps to preserve blood pressure following hemorrhagic shock, potentially supporting the maintenance of organ function.

**FIGURE 3:**
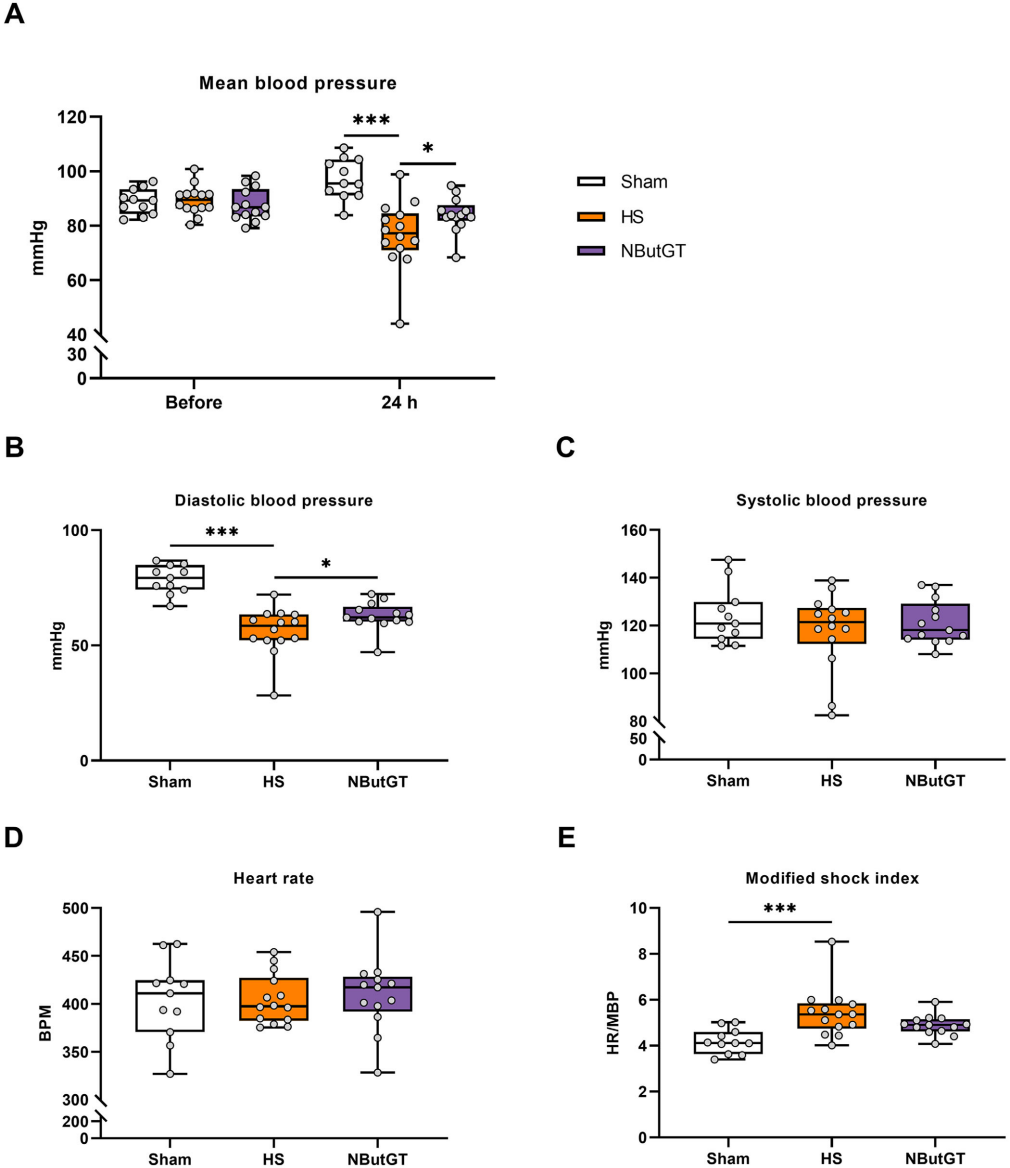
NButGT improves arterial blood pressure. (A) Mean blood pressure before and 24 h after the hemorrhagic shock. (B) Diastolic, (C) systolic blood pressure, (D) heart rate, (E) and modified shock index 24 h after the hemorrhagic shock. The sham group underwent surgery only. Hemorrhagic shock group underwent hemorrhagic shock protocol. NButGT group underwent hemorrhagic shock with dual injection of NButGT at 10 mg/kg. Statistical analyses were assessed by two‐way ANOVA (A), Kruskal–Wallis test (C and E) or one‐way ANOVA (B and D). HS: hemorrhagic shock. *: *p* < 0.05; ***: *p* < 0.001. *n* = 11–14.

### 2.4 Improvement of Mean Arterial Pressure Reduces Organ Injury

Shock is defined by a multiorgan dysfunction, which, if not promptly managed, leads to a rapid death. To assess whether improved hemodynamics translated into preserved organ function, plasmatic markers of organ injury were measured 24 h after hemorrhagic shock. Lactatemia is an indicator of systemic metabolic alteration and was increased in the hemorrhagic shock group (Sham: 1.15 ± 0.40; HS: 2.15 ± 1.03; mmol/L; *p* < 0.01). Twenty‐four hours post‐shock, NButGT treatment had no impact on this parameter (Figure 4A). The base excess, calculated from bicarbonate levels and pH, has been shown as a mortality predictor in traumatic patients [41]. Most traumatic patients with hemorrhagic shock have a severe deficit in base excess. The base excess was reduced in the hemorrhagic shock group (Sham: 8.11 ± 2.73; HS: 4.83 ± 2.07; mmol/L; *p* < 0.01) and restored with the NButGT treatment (NButGT: 8.11 ± 2.54; mmol/L; *p* < 0.05), mainly by increasing the bicarbonate concentration, while the arterial pH was unchanged between the different groups (Figure 4B, Figure S1). Plasmatic levels of creatinine and cardiac troponin T are markers of renal and cardiac dysfunction, respectively [42, 43]. Both were increased in the hemorrhagic shock group (creatininemia: Sham: 20.1 ± 1.0; HS: 30.4 ± 24.7; µmol/L; cardiac troponin T: Sham: 14.3 ± 5.3; HS: 148.1 ± 205.4; ng/L; *p* < 0.05) and partly restored by NButGT treatment (creatininemia: NButGT: 24.0 ± 2.9; µmol/L; cardiac troponin T: NButGT: 40.2 ± 30.5; ng/L; ns) (Figure 4C,D). The plasma levels of alanine amino‐transferase (ALAT) and aspartate amino‐transferase (ASAT) correlate positively with hepatocellular injury [44]. ALAT and ASAT plasma levels were increased in the hemorrhagic shock group (ALAT: Sham: 56.1 ± 27.0; HS: 264.7 ± 220.2; ASAT: Sham: 356 ± 249; HS: 1339 ± 729; UI/L; *p* < 0.001). NButGT reduced ALAT and ASAT plasma levels by 26% and 28%, respectively (ALAT: NButGT: 194.3 ± 165.8; ASAT: NButGT: 961 ± 450; UI/L; ns) (Figure 4E,F). These data indicate that multiple organs are affected in the hemorrhagic shock group, while NButGT treatment leads to an overall reduction in markers of organ injury.

**FIGURE 4:**
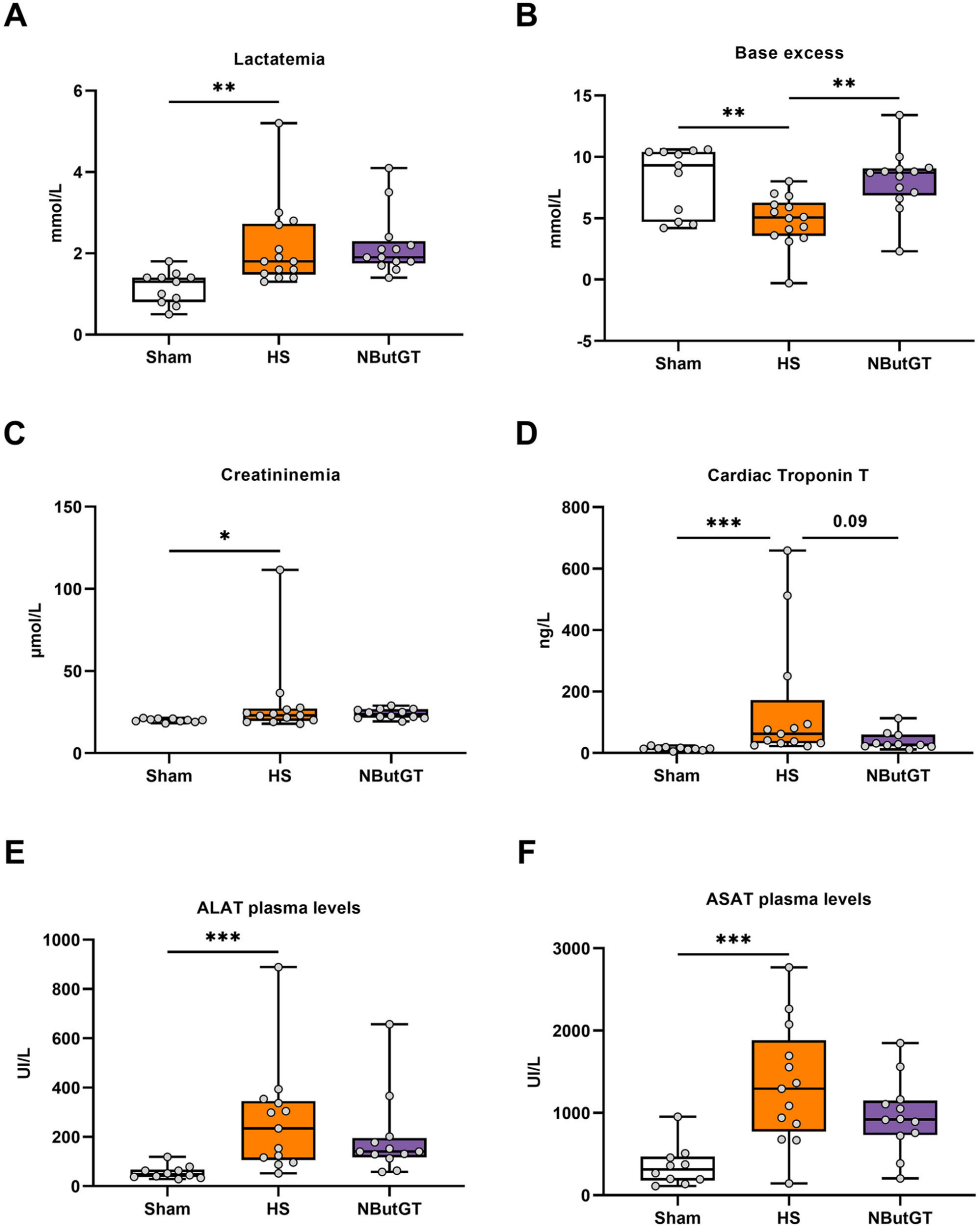
Stimulation of O‐GlcNAcylation limits organ alteration. Quantification of (A) arterial lactate, (B) base excess, (C) creatininemia, (D) cardiac troponin T, (E) ALAT, and (F) ASAT plasmatic levels 24 h after the hemorrhagic shock. The sham group underwent surgery only. Hemorrhagic shock group underwent hemorrhagic shock protocol. NButGT group underwent hemorrhagic shock with dual injection of NButGT at 10 mg/kg. Statistical analyses were assessed by Kruskal–Wallis test (A, C, D, and E) or one‐way ANOVA (B and F). HS: hemorrhagic shock. *: *p* < 0.05; **: *p* < 0.01; ***: *p* < 0.001. *n* =  11–14.

### 2.5 NButGT Restores Sodium and Potassium Balance

Hemorrhagic shock impairs cellular membrane function, compromising the regulation of ion exchange between the intracellular and extracellular environments [45]. We assessed the concentrations of different ions under our experimental conditions. Blood concentrations of calcium and chloride were unchanged between the different groups (Figure 5A,B). Natremia was decreased, whereas kalemia was increased in the hemorrhagic shock group (natremia: Sham: 141.6 ± 1.3; HS: 139.7 ± 1.8; kalemia: Sham: 4.17 ± 0.29; HS: 4.60 ± 0.53; mmol/L; *p* < 0.05). NButGT treatment restored both natremia and kalemia (natremia: NButGT: 141.9 ± 1.6; kalemia: NButGT: 4.28 ± 0.44; mmol/L; *p* < 0.01) (Figure 5C,D). These findings suggest that NButGT may affect proteins involved in the regulation of sodium and potassium homeostasis.

**FIGURE 5:**
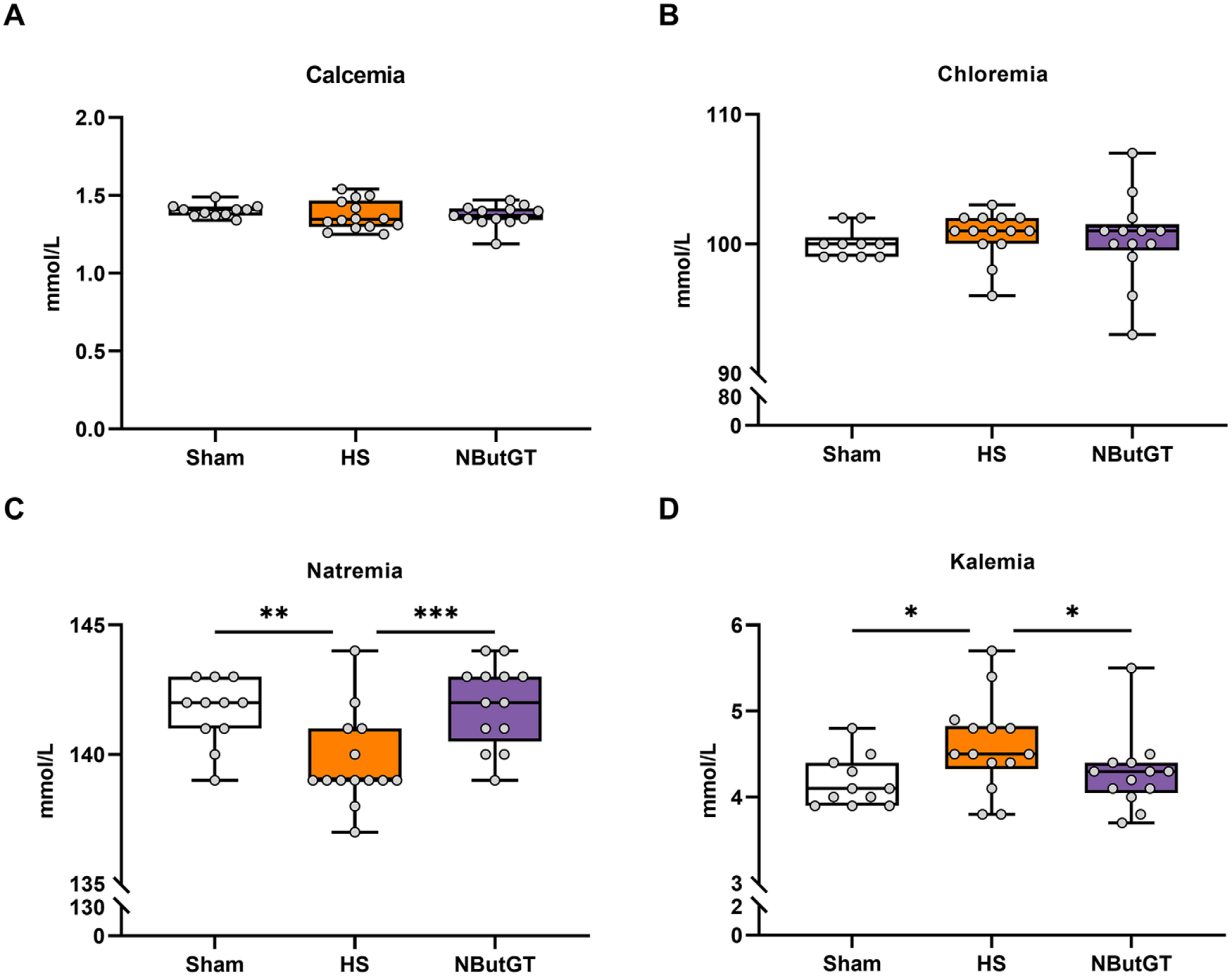
Ionic balance is altered by hemorrhagic shock and restored with O‐GlcNAcylation stimulation. Quantification of (A) calcemia, (B) chloremia, (C) natremia and (D) kalemia 24 h after the hemorrhagic shock. The sham group underwent surgery only. Hemorrhagic shock group underwent hemorrhagic shock protocol. NButGT group underwent hemorrhagic shock with dual injection of NButGT at 10 mg/kg. Statistical analyses were assessed by one‐way ANOVA (A and C) or Kruskal–Wallis test (B and D). HS: hemorrhagic shock. *: *p* < 0.05; **: *p* < 0.01; ***: *p* < 0.001. *n* = 10–14.

### 2.6 The Na^+^/K^+^ ATPase Is O‐GlcNAcylated

Considering that the Na^+^/K^+^ ATPase (NKA) is the pump responsible for the entry into the cells of 2 K^+^ ions and the extrusion of 3 Na^+^ ions and that NKA has putative O‐GlcNAcylation sites, the next step was to validate if NKA is effectively O‐GlcNAcylated in sham condition. An immunoprecipitation followed by a Wheat Germ Agglutinin (WGA)‐based lectin affinity gel electrophoresis was performed to evaluate the O‐GlcNAc status of NKA. The appearance of a >270 kDa band confirms that NKA was retained during migration on the WGA gel hence is O‐GlcNAcylated (Figure 6A). This observation was further confirmed using an anti‐O‐GlcNAc antibody, validating NKA O‐GlcNAcylation (Figure 6B).

**FIGURE 6:**
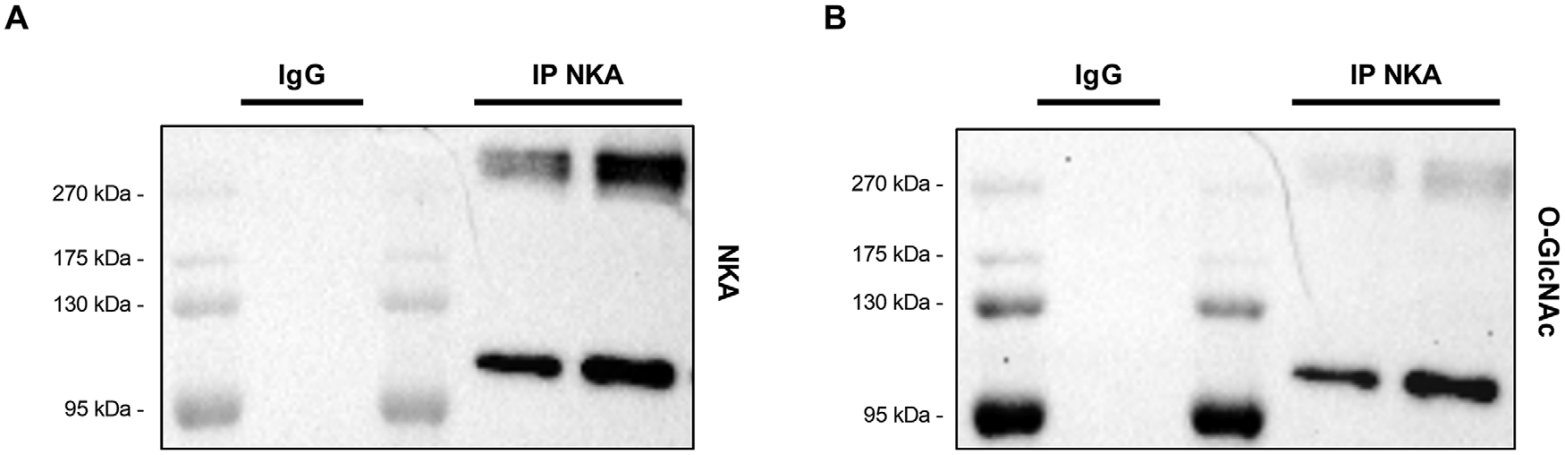
Evaluation of the Na^+^/K^+^ ATPase O‐GlcNAcylation. Western blot illustrating (A) NKA or (B) O‐GlcNAcylation. Renal proteins from sham rats were immunoprecipitated with an NKA antibody (IP NKA) or a non‐specific antibody (IgG). Data shown are representative of two independent experiments.

### 2.7 Na^+^/K^+^ ATPase Expression and Activity are Impacted by NButGT Treatment

After demonstrating that Na^+^/K^+^ ATPase was O‐GlcNAcylated, we wanted to better understand its impact on NKA. As NKA expression and activity is mostly found in kidneys and heart, we evaluated the impact of the early O‐GlcNAc stimulation with the NButGT treatment on NKA renal and cardiac expression. Cardiac NKA expression remained unchanged in the different groups, whereas renal NKA expression was reduced following hemorrhagic shock (Sham: 14.7 ± 3.5; HS: 10.9 ± 2.2; % of area; *p* < 0.01) (Figure S2, Figure 7A). The O‐GlcNAcylation stimulation restored NKA expression in kidneys (NButGT: 15.0 ± 3.8; % of area; *p* < 0.01) (Figure 7A). The restoration of natremia and kalemia by NButGT suggests that O‐GlcNAcylation may affect NKA membrane addressing or its activity. PAN‐CADHERIN and GAPDH were used as markers to confirm the successful separation of membrane and cytosolic fractions, respectively. Renal cytosolic and membrane expression levels of NKA did not show significant changes following hemorrhagic shock or NButGT treatment (Figure 7B). To investigate the impact of O‐GlcNAcylation on NKA activity, HEK 293 cells were treated with NButGT. After 24 h of treatment, NButGT did not affect cell viability and effectively increased O‐GlcNAcylation levels in HEK 293 cells, while total ATPase activity remained unchanged (Figures S3 and S4; Figure 7C). To evaluate the specific contribution of NKA to overall ATPase activity, cells were treated with ouabain, a specific NKA inhibitor. Cotreatment with ouabain and NButGT resulted in a significant reduction in ATPase activity compared to ouabain alone, indicating that NButGT enhanced NKA activity (ouabain alone: 0.72 ± 0.21; ouabain + NButGT: 0.44 ± 0.11; *p* < 0.05) (Figure 7C). Together, these results underscore that NButGT modulates both expression and activity of the NKA.

**FIGURE 7:**
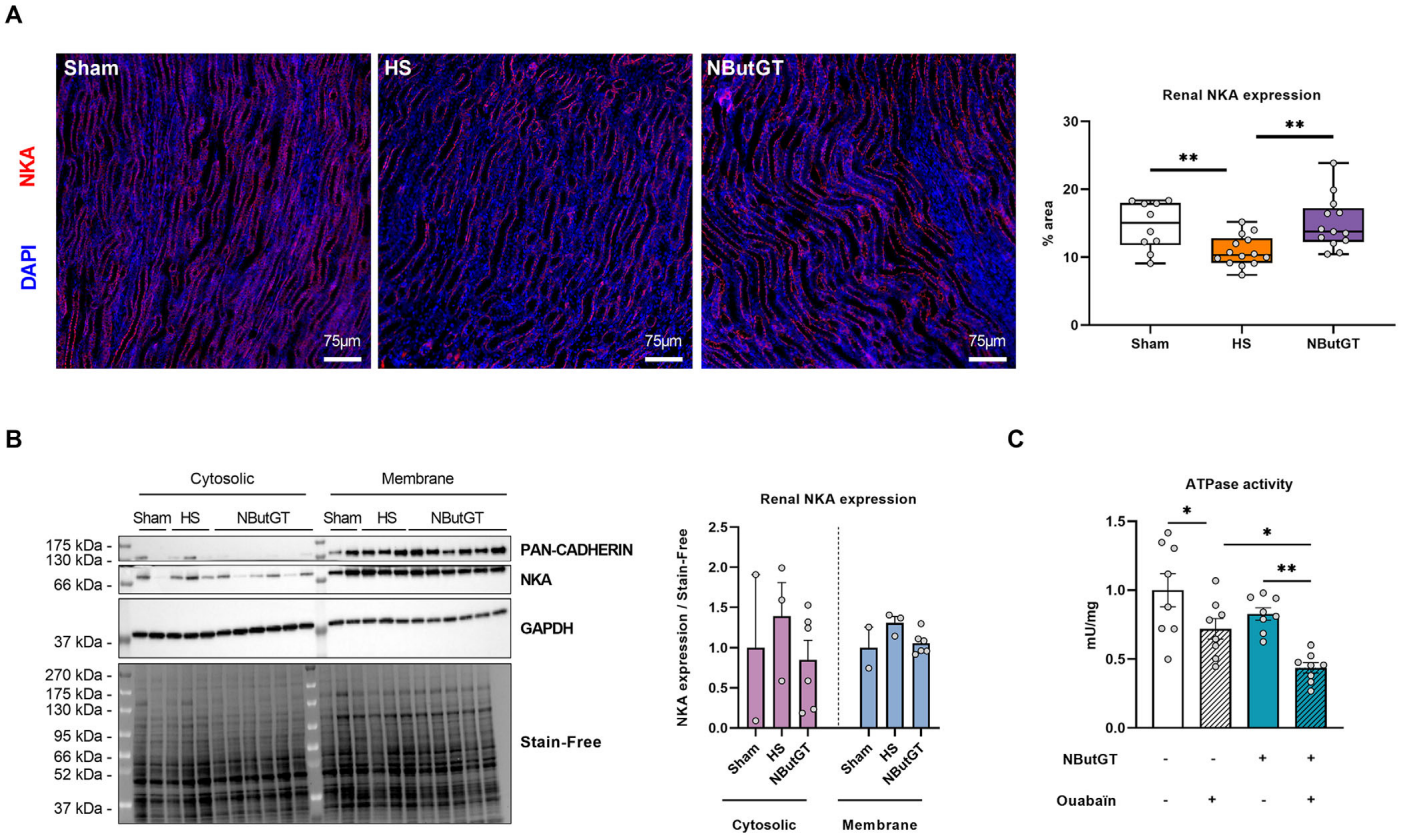
NButGT modulates Na^+^/K^+^ ATPase expression and activity. (A) Evaluation of Na^+^/K^+^ ATPase pump expression in the kidney of sham, hemorrhagic shock or NButGT treated rats 24 h after the hemorrhagic shock (*n* = 10–14). (B) Evaluation of the renal cytosolic and membrane Na^+^/K^+^ ATPase pump expression of sham, hemorrhagic shock or NButGT treated rats 24 h after the hemorrhagic shock (*n* = 2–6). (C) Measurement of ATPase activity in HEK 293 cells with or without NButGT (10 µM) and/or ouabain (100 µM) treatments (*n* = 8). The sham group underwent surgery only. Hemorrhagic shock group underwent hemorrhagic shock protocol. NButGT group underwent hemorrhagic shock with dual injection of NButGT at 10 mg/kg. Statistical analyses were assessed by one‐way ANOVA (A and C) or two‐way ANOVA (B). HS: hemorrhagic shock. *: *p* < 0.05; **: *p* < 0.01.

## 3 Discussion

In this study, we demonstrated for the first time that the acute systemic stimulation of O‐GlcNAcylation via NButGT treatment improves the overall status of rats 24 h after a hemorrhagic shock through an improvement of hemodynamic parameters and electrolyte imbalance. We identified one of the mechanisms associated with the beneficial effect through a modulation of the renal Na^+^/K^+^ ATPase expression and activity.

In our model, hemorrhagic shock does not alter the protein expression of either OGT or OGA in the heart. Interestingly, both GFAT1 and GFAT2 cardiac protein levels are increased in response to hemorrhagic shock. Although these two enzymes are encoded by distinct genes (*GFPT1* and *GFPT2*) and share 75% sequence homology, their expression patterns differ throughout development and across tissues, suggesting that they fulfill distinct biological functions [46, 47, 48]. At the cardiac level, both GFAT isoforms are expressed, although the predominance of one isoform over the other has not yet been clearly established [49, 50]. Hemorrhagic shock induces nutrient deprivation and endoplasmic reticulum stress, activating the unfolded protein response (XBP1, ATF4, HSPs), while associated ischemia‐reperfusion generates systemic hypoxia and reactive oxygen species production (ROS) [51, 52]. GFAT1 has been reported to be induced by the spliced form of XBP1 and ATF4, enhancing hexosamine biosynthetic pathway flux and protein O‐GlcNAcylation levels, thereby leading to neuro‐ and cardioprotection in brain injury after subarachnoid hemorrhage and ischemia–reperfusion [53, 54, 55]. GFAT2, strongly induced by hypoxia via hypoxia‐inducible factor‐1 alpha [49, 56], is also upregulated by oxidative stress—protecting against ROS toxicity [57, 58]—and by lipopolysaccharide through TLR4 pathways activated by oxidative stress and endogenous damage‐associated molecular patterns from tissue injury [52, 59, 60, 61]. Taken together, this may help explain, at least in part, the increase in both GFAT1 and GFAT2 protein expression observed in our model of hemorrhagic shock. Interestingly, despite this increase, global O‐GlcNAcylation levels remain unchanged in the heart, suggesting the existence of a feedback control mechanism. NButGT treatment, by inhibiting OGA, robustly increased O‐GlcNAcylation levels compared with hemorrhagic shock alone. These findings suggest that pharmacological OGA inhibition synergizes with stress‐induced HBP activation, thereby improving outcomes during hemorrhagic shock.

Base excess, a marker of injury severity, correlates with patient outcomes in hemorrhagic shock [62, 63, 64] and is decreased in our model of hemorrhagic shock at 24 h. Applied at the early phase of shock, NButGT treatment restores the bicarbonatemia and base excess. Our model of hemorrhagic shock is associated with a multiorgan failure characterized by an elevation of different markers such as ASAT, ALAT, creatinine and cardiac troponin T levels. Interestingly, we demonstrated that NButGT treatment improves all these parameters. These results are consistent with the benefit of the O‐GlcNAcylation stimulation on the organ blood flow during trauma hemorrhage [31, 33]. Overall, our results strengthen studies which demonstrated that glutamine (metabolite essential for the UDP‐GlcNAc synthesis, the OGT sugar donor) administration ameliorates tissue injury in hemorrhagic shock model and that glutamine supplementation in patients with multiple trauma limits infectious complications and reduces mortality [65, 66, 67, 68]. In clinic, restoration of a normal blood pressure is the primary objective in the management of the hemorrhagic shock [69]. We confirmed that the drop in blood pressure is efficiently reduced with the NButGT treatment at the early phase of the shock situation. We and others have previously reported that the O‐GlcNAc stimulation restores the mean blood pressure in both young and adult rats in septic shock and after trauma hemorrhage protocol [25, 26, 31, 32, 70]. Interestingly, Shires et al. demonstrated that the drop in blood pressure was associated with a depolarization of muscle cells in hemorrhagic shock primates, suggesting an involvement of ionic transports in blood pressure regulation during hemorrhagic shock [71].

In our model of hemorrhagic shock, and as described in Human [71, 72, 73, 74], we report a decrease in natremia and an increase in kalemia. The increase in kalemia has been shown to be a marker of tissue hypoxia, and correlates with mortality [75, 76, 77]. It has been demonstrated that the occurrence of electrolyte movements results in cellular depolarization that progresses in a sequential manner throughout the course of hemorrhagic shock. The consequence of this cellular depolarization is an amplification of the volume loss from the vasculature, thereby imposing limitations on the effectiveness of cardiovascular stabilization efforts [78]. Eastridge et al. suggested that a “poisoning” of NKA is associated with the modification of Na^+^, K^+^ and Ca^2+^ concentrations, contributing to cellular depolarization, independently of oxygen delivery or energy availability [78]. We, and others, have identified using mass‐spectrometry approaches that NKA is potentially O‐GlcNAcylated in rat, mice or human [26, 79, 80, 81, 82, 83, 84, 85, 86]. By using a WGA‐based lectin affinity gel electrophoresis, we have validated that NKA is O‐GlcNAcylated, which is consistent with a recent study which revealed O‐GlcNAcylation of NKA in LLC‐PK1 kidney cells [87]. Prompted by this result, we wanted to evaluate the impact of O‐GlcNAcylation on renal NKA. Via histological analysis, we have highlighted that O‐GlcNAcylation stimulation restores renal NKA expression in hemorrhagic shock situation and that NButGT treatment increases its activity. Abdel‐Zaher et al. demonstrated that treating rabbits in hemorrhagic shock with adenosine reduced mortality and improved cardiac contractility and ionic balance, yet they did not identify the underlying mechanism [72]. These beneficial effects may involve NKA as Darlington et al. demonstrated that the stimulation of NKA in erythrocyte with adenosine and other purine nucleosides was associated with an improvement of rat survival during hemorrhagic shock. They also observed an improvement of the mean arterial pressure and a return to the kalemia baseline [73, 74]. Moreover, they demonstrated that inhibition of NKA contributes to shock and death [88]. In our model, the NButGT treatment is associated with a decrease in creatininemia which suggests an alleviation of the kidney injury. Zhang et al. demonstrated that NKA activity was altered during hemorrhagic shock, and concluded that ATPase failure during hemorrhagic shock contributed to kidney injury [89]. Our findings demonstrate that NButGT enhances NKA activity and restores its expression, revealing a potential mechanistic pathway underlying its renal protective effects. These benefits may also involve additional mechanisms, given that previous studies demonstrated that glucosamine, which promotes O‐GlcNAcylation, attenuates oxidative stress and apoptosis and improves glomerular dysfunction in kidney injury model [90, 91]. Beyond its renal effects, the impact of NButGT on NKA could also contribute to the cardioprotective outcomes observed in our study. In fact, NKA function and/or expression are reduced in heart failure, and its stimulation has been shown as protective in ischemia–reperfusion [92, 93]. Moreover, NKA activators have been reported to reduce arrhythmia in the context of ischemic heart injury [94, 95].

In summary, we demonstrate that O‐GlcNAcylation stimulation at the early phase of hemorrhagic shock improves outcomes, restores renal Na^+^/K^+^ ATPase pump expression and electrolyte imbalance. Overall, we highlight that stimulation of O‐GlcNAcylation may be a novel therapeutic strategy at the early phase of shock to reduce the hemorrhagic shock burden. This is all the more promising as we have recently demonstrated the safety of NButGT on healthy anesthetized rats [96]. It would be valuable to establish a cohort of patients suffering from hemorrhagic shock to monitor O‐GlcNAcylation levels throughout the clinical course to consider the potential development of a new, non‐symptomatic, approach of the treatment of hemorrhagic shock.

The main limitation of this study is its exclusive focus on male rats. The use of males was chosen to minimize biological variability arising from estrous cycle‐dependent fluctuations in hormonal and metabolic status, which can influence cardiac function, stress responses, and O‐GlcNAc signaling [97, 98, 99]. Notably, cardiac O‐GlcNAcylation levels have been described to be higher in females in a pressure overload–induced hypertrophy model, and both OGT and OGA display sex dependent regulation [100]. This is further supported by evidence that higher OGT activity and elevated O‐GlcNAcylation in female murine hearts are associated with reduced susceptibility to ischemic injury compared with males [101]. In light of these findings, it is recognized that the effects of O‐GlcNAcylation modulation described in the present work may differ in females, potentially resulting in a more pronounced protective phenotype or in sex specific mechanisms. Accordingly, future studies will be required to specifically evaluate the impact of sex on these O‐GlcNAc‐dependent effects.

## 4 Material and Methods

### 4.1 Reagents

O‐GlcNAcase inhibitor NButGT was synthetized as described by Matthew S. Macauley [102].

### 4.2 Hemorrhagic Shock Model

Male Wistar Han rats (Charles River, Saint‐Germain‐Nuelles, France) were delivered to the Unité Thérapeutique Expérimentale at 10 weeks of age. Rats were housed for at least 1 week to acclimatize under standard conditions: temperature (21°C–24°C), humidity (40%–60%), and a 12‐h light/dark cycle with the light phase starting at 07:00 AM. Food and water were available ad libitum. Experiments were performed in accordance with the ethics committee in charge of animal experimentation of the Pays de la Loire, French law on animal welfare, EU Directive 2010/63/EU for animal experiments (#17858 and #32499), and the National Institutes of Health (NIH) Guide for the Care and Use of Laboratory Animals (NIH Pub. No. 85‐23, revised 2011). The reporting is in accordance with current ARRIVE guidelines 2.0.

The hemorrhagic shock protocol was performed as previously described [103]. Eleven‐ to thirteen‐week‐old rats were anesthetized with a mixture of air and sevoflurane (Sevotek, Axience, Pantin, France) (induction: 8% sevoflurane, flow rate 1 L/min; maintenance during the surgical procedure: 4% sevoflurane, flow rate 0.6 L/min; maintenance after the surgical procedure: 3% sevoflurane, flow rate 0.6 L/min). Rats were shorn and sanitized in the surgical areas (inguinal and neck regions) and placed in *dorsal decubitus* on a heating mat thermoregulated via a rectal probe to maintain their temperature within physiological values (37.5°C–38.5°C).

The right jugular vein and left femoral artery were cannulated using PE‐50 and PE‐10 tubing, respectively (PHYMEP, Paris, France). The venous cannulation allows administration of vascular filling while the arterial cannulation allows hemodynamic monitoring and blood sampling. A no touch, 10‐min period, after induction and catheterization allowed a stabilization of hemodynamic parameters before measuring pre‐protocol values for 5 min. Basal mean blood pressure below 80 mmHg was considered an exclusion criterion. All rats received were included in the study.

The model of hemorrhagic shock is a mixed model comprising a fixed‐volume hemorrhage phase followed by a fixed‐pressure hemorrhage phase. The first period corresponds to a sampling of 5 mL of blood in 5 min to mimic the loss of a large volume of blood. During the second period, the mean blood pressure was maintained between 32 and 38 mmHg for 75 min. If the mean blood pressure exceeded 38 mmHg, 200 µL of blood was collected. On the contrary, if the mean blood pressure was lower than 32 mmHg, 200 µL of a mixture composed of 50% blood and 50% Ringer Lactate was injected (Fresenius Kabi, Bad Homburg, Germany). The hemorrhagic shock period was followed by a fluid resuscitation period with an intravenous injection of Ringer Lactate at room temperature (10 mL/kg) over a 20‐min period. At the end of the resuscitation phase, animals were sutured, injected with buprenorphine at 0.05 mg/kg (Buprécare, Axience, Pantin, France) and placed back in their housing room.

Sixteen hours after surgery procedure, animals were injected with buprenorphine at 0.05 mg/kg. Twenty‐four hours after surgery procedure, animals were anesthetized and placed on a heating mat as described above. The left carotid artery was cannulated to evaluate the 24‐h hemodynamic values. Lactate measurement was performed on a drop of blood from the tail with StatStrip Xpress Meter (Nova Biomedical, Les Ulis, France). A 10‐min period was observed before recording the hemodynamics values.

Rats were randomly assigned to each group. Only the experimenter was aware of the group allocation at the different stages of the experiment. The NButGT and hemorrhagic shock groups have undergone exactly the same procedures. A subcutaneous injection of NButGT, an OGA inhibitor (10 mg/kg; 0.2 mL/kg; NButGT group; *n* = 13) or Ringer's Lactate (0.2 mL/kg; hemorrhagic shock group; *n* = 14) was administered at the end of the first sampling period and 16 h post‐surgery. The Sham group has undergone surgery only, without stages of exsanguination, hemorrhagic shock and resuscitation (n = 11) (Figure S5). No confounding factors were identified in this study. No expected or unexpected adverse effects were observed during the study.

### 4.3 Organs and Blood Sampling

After recording hemodynamic values at 24 h, arterial blood was collected from the carotid artery to measure blood gas electrolyte and metabolite with Epoc blood analysis system (Siemens Healthineers, Erlangen, Germany) [104]. Arterial blood was collected from the carotid artery of rats, and plasma was separated in heparinized tubes by centrifugation at 1600 g for 10 min and stored at −80°C until biochemical analysis (please referred to Section 4.6). Heart and kidneys were harvested under standardized conditions (between 4:00 PM and 5:00 PM) to avoid circadian modifications on protein O‐GlcNAcylation levels and were immediately frozen in liquid nitrogen for biochemical analyses or included in 4% paraformaldehyde or Tissue‐Plus O.C.T. compound (#4585, fisher scientific) for histological analyses.

### 4.4 Tissue Preparation and Protein Extraction

Frozen tissues were crushed in a mortar to obtain a powder that will be used for protein analyses. To preserve post‐translational modifications, all grinding steps were carried out in liquid nitrogen. Powder was then stored at ‐80°C. Extraction for proteins from tissues or cells was realized as previously described [46]. Membrane proteins from rat kidney were extracted by using Mem‐PER Plus Membrane Protein Extraction Kit (#89842, ThermoFisher, Waltham, Massachusetts, United States of America) according to the manufacturer's instructions.

### 4.5 Western Blot

Western blotting experiments were performed as previously described [105]. The antibodies used are listed in Table S1. The amount of protein used for Western blot was 25 µg. Analyses were performed using Image Lab software (Image Lab 6.1 version, Bio‐Rad, CA, United States of America). Full unedited blots are shown in Figure S6.

### 4.6 Biochemical Assays

Aspartate amino‐transferase (ASAT) and alanine amino‐transferase (ALAT) levels were determined using international federation of clinical chemistry enzymatic method with pyridoxal phosphate activation using Cobas c303 (Roche, Meylan France) [106, 107]. Creatininemia was determined by enzymatic method (creatininase) using Cobas c303 and high‐sensitivity cardiac Troponin T levels were determined using electrochemiluminescent immunoassay (ECLIA) using Cobas e402 (Roche, Meylan, France) [108, 109]. All assays were performed using Roche's dedicated reagents, calibrators, and controls. Independent internal (Bio‐Rad) and external (Randox, Crumlin, United Kingdom) controls were also used to ensure the accuracy and precision of the assays. All biochemical measurements were performed in the laboratory of Biochemistry, CHU Nantes, Laënnec Hospital, France.

### 4.7 Immunoprecipitation

Immunoprecipitation was performed according to a previously described protocol [110]. Beads (Dynabeads, 10003D, Invitrogen, Waltham, Massachusetts, United States of America) were placed in a 1.5 mL tube on a magnetic holder (25 µL of beads per condition). Then, they were washed twice with 1 mL of PBS 1X (Phosphate Buffered Saline, pH 7.4, #14190‐169, Invitrogen, Waltham, Massachusetts, United States of America). Anti‐Na^+^/K^+^ ATPase antibody (#ab7671, Abcam, Cambridge, United Kingdom) was added at 4 µg except for the control tube where it was replaced by an isotype‐matched control antibody from the same species (#31160, ThermoFisher, Waltham, Massachusetts, United States of America). Beads were then suspended in their original volume with PBS 1X and incubated for 40 min at room temperature with slow agitation to bind the antibody to the beads. The supernatant was removed and washed three times with 1 mL of PBS 1x containing 0.1% Tween 20 and two times with 1 mL of 0.2 M triethanolamine (pH = 8.2). Supernatant was resuspended in 1 mL of dimethyl pimelimidate solution (54 mg dimethyl pimelimidate in 10 mL of 0.2 M triethanolamine, pH = 8.2) and placed under slow rotation for 30 min. Supernatant was then removed and beads were incubated with 1 mL of 50 mM Tris (pH = 7.5) under slow rotation for 15 min at room temperature. To enhance bead/antibody interactions, the beads were sequentially washed with: PBS 1X, 0.1 M citrate buffer (pH = 3.1), 0.1 M sodium phosphate buffer, PBS 1X (twice) and PBS 1X containing 0.5% sodium deoxycholate (NaDOC). Beads were then incubated with NaDOC for 15 min under slow rotation at room temperature. They were washed twice with PBS 1X containing 0.1% Tween 20 and then three times with the lysis buffer previously described in Section 4.4. Once the beads were dry, they were suspended in their initial volume (25 µL) in lysis buffer.

Protein extracts from the kidney samples were added to the beads (2 mg/condition). Samples were incubated under slow rotation for 2 h at 4°C. Then, tubes were washed three times with lysis buffer. At the last wash, when the tube was dry, 5 µL of NuPAGE LDS Sample Buffer (4X) (NP0007, Invitrogen, Waltham, Massachusetts, United States of America) and 26 µL of water was added to each tube and then heated for 10 min at 60°C to elute the proteins from the beads. Then, 4 µL of NuPAGE Sample Reducing Agent (10X) (NP0004, Invitrogen, Waltham, Massachusetts, United States of America) was added. The supernatant containing the immunoprecipitated protein was recovered for further experiments.

### 4.8 Wheat Germ Agglutinin‐based Lectin Affinity Gel Electrophoresis

A Wheat Germ Agglutinin (WGA) gel was realized to study the O‐GlcNAcylated forms of NKA according to the composition shown in Table S2, as previously described [111, 112]. Each part of the gel was prepared extemporaneously. Before adding the 10% APS, a 15 min degassing period was performed to promote polymerization. The 10 mg/mL WGA solution was prepared with 10 mM of Tris/HCl buffer (pH 7.5) and stored at −20°C. Migration was realized at constant amperage (25 mA). The next steps were performed as described previously in Section 4.5.

### 4.9 ATPase and Na^+^/K^+^ ATPase Activity

ATPase and Na^+^/K^+^ ATPase activity were assessed on human embryonic kidney 293 (HEK 293) cells by using the ATPase Assay Kit (#ab270551, Abcam, Cambridge, United Kingdom) following the manufacturer's instructions. HEK 293 cells were cultured in Dulbecco's modified Eagle's medium (#41966‐029, Gibco, France) supplemented with 10% fetal bovine serum (#FB‐1001, Biosera, Cholet, France), 4.5 g/L glucose, 2 mmol/L L‐glutamine, 100 U/mL penicillin and 100 µg/mL streptomycin (#25030‐024, #15140‐122, ThermoFisher, United Kingdom) at 5% CO_2_, in a humidified incubator maintained at 37°C. The cell line was confirmed to be mycoplasma‐free (#LT07‐318, MycoAlert, Lonza, France). To evaluate the impact of O‐GlcNAcylation, cells were pretreated with 10 µM NButGT 24 h before the activity assay. To measure the relative activity of the Na^+^/K^+^ ATPase, 100 µM of ouabain (#O0200000, Sigma‐Aldrich, Saint‐Louis, Missouri, United States of America), a specific inhibitor of the Na^+^/K^+^ ATPase, was added just before the activity assay.

### 4.10 Histology

Kidneys were harvested and immediately fixed in 4% paraformaldehyde solution for a minimum of 24 h at 4°C. After fixation, samples were transferred into processing cassettes and subjected to a graded alcohol dehydration series. Initial dehydration steps consisted of two incubations in 70% ethanol at 70°C for 15 min each, followed by a third incubation in 80% ethanol at 80°C for 1 h. Subsequently, cassettes were placed in a tissue processor (Microm STP 120 Spin Tissue Processor, ThermoFisher, United States of America), and the following automated processing protocol was applied, with a change of solution between each incubation: one bath of 80% ethanol at 80°C for 1 h; three baths of 95% ethanol: 1 h, 1 h 30 min, and 1 h 30 min; three baths of 100% ethanol: 1 h 15 min, 1 h 30 min, and 2 h; three baths of butanol: 2 h, 2 h 30 min, and 2 h 30 min; two baths of paraffin: 2 h 30 min and 2 h. After processing, each tissue sample was positioned in a 22 × 22 mm^2^ plastic mold (#2219, ThermoFisher, United States of America) previously filled with a thin layer of molten paraffin. The sample was then fully covered with paraffin and placed onto a cooling plate set at −15°C (Micron Microtech EC 350–2, ThermoFisher, United States of America) until complete solidification. Samples were then store at −4°C. Kidney sections (7 µm thick) were cut using a microtome (Microm HM 355S, ThermoFisher, United States of America). Then, kidney sections were de‐paraffinized three times in Tissue‐Tek Tissue‐Clear (#1466, Sakura Finitek, United States of America) and immersed in 100%, 95%, 80%, 70% alcohol baths. Slides were placed in a water bath of 10 mM citrate buffer at 90°C for 40 min. Normal donkey serum blocking buffer (#NB‐23‐00183‐2, Neo Biotech, France) was then placed on each kidney section for 30 min. Slides were incubated with anti‐Na^+^/K^+^ antibody (1:500, #ab7671) overnight in a wet chamber at 4°C. Slides were washed three times in PBS 1X before incubating with Alexa Fluor 647 anti‐mouse antibody (#A31571, Life technologies, United States of America) for 1 h at room temperature. Slides were washed three times with PBS 1X and coated with Fluoromount mounting medium coupled to DAPI (#0100‐20, SouthernBiotech, Birmingham, United States of America). Slides were placed at 37°C for 5 h and then at 4°C until observation. They were imaged using a Nikon Eclipse Ti2 inverted. All images were obtained using a 20× objective and were analyzed using Image J software (V1.53U version).

Hearts were collected and immediately embedded in OCT compound, then sectioned transversely at 7 µm using a cryostat (CryoStar NX70). Sections were brought to room temperature, air‐dried, and fixed in 4% paraformaldehyde. Antigen retrieval was performed in preheated citrate buffer (10 mM, pH 6) at 90°C. After blocking with 5% normal donkey serum in PBS, sections were incubated overnight with a primary antibody against Na^+^/K^+^‐ATPase (2 µg/mL, #ab7671). Following primary incubation, sections were incubated for 1 h with a donkey anti‐mouse Alexa Fluor 647‐conjugated secondary antibody (2.5 µg/mL, #A31571). The sections were then mounted using a DAPI‐containing mounting medium and coverslipped. Images were acquired using a Nikon Eclipse Ti2 inverted microscope equipped with a Nikon DS‐Qi2 digital camera using a 40× objective and were analyzed using Image J software (V1.53U version).

### 4.11 Cell Viability

Cells were seeded at 650,000 cells per well in 6‐well plate. After 24 h, cells were treated with 0.1, 1, 10 or 100 µM of NButGT for 24 h or 1 µM of staurosporine (#S4400, Sigma‐Aldrich) for 2 h. Cells were detached using accutase (#07922, Stemcell) and stained with propidium iodide (2.5 µg/mL) (#P1304MP, ThermoFisher Scientific) for 15 min at room temperature. Then, 50,000 cells per condition in duplicate, were acquired on a Canto II flow cytometer (BD Biosciences). Data were analyzed using FlowJo software (version 10.10).

### 4.12 Statistical Analysis

Results are expressed as mean ± standard deviation of *n* biological replicates. Analyses of Western blots are expressed in relation to the average of the protein quantification (Stain‐Free) and then reduced to the average of the control samples (sham or untreated condition). The normal distribution of the data was tested with D'Agostino & Pearson test. If the distribution of the data was normal, data were analyzed by a one‐way ANOVA with Fisher's LSD test; if not, data were analyzed by a Kruskal–Wallis test with uncorrected Dunn's test. Group comparisons with more than two variables were analyzed with a two‐way ANOVA with Fisher's LSD test. A *p*‐value of less than 0.05 was considered statistically significant. All statistical analyses and graphs were performed using GraphPad Prism software (version 8.4.2).

## Author Contributions

T.D., V.A., B.R., M.D., and B.L. contributed to conceptualization. T.D., A.V., V.A., M.B., A.E., T.P., A.P., B.R.O., J.M., A.G., J.Lec., N.T., M.G., A.B., M.R., J.Leb., A.T., E.B., A.A.L., and N.J.P. contributed to the investigation. T.D. and B.L. prepared the original draft of the manuscript. T.D., A.V., B.R., M.D., and B.L. contributed to writing – review and editing. M.D.W., B.R., and B.L. contributed to funding acquisition. All authors have read and approved the final manuscript.

## Funding

This work was supported by the “Agence Nationale de la Recherche” (grant no.: 20ASTC003201hErOiSmE) (Paris, France) and the “Direction Générale de l’Armement” (Paris France). These funders provided overall project funding and had no role in study design, data collection and analysis, interpretation of results, manuscript preparation, or the decision to submit for publication.

T.D. was supported by grants from Direction Générale de l'Armement, France and Région des Pays de la Loire, France during his PhD. A.P. was supported by grants from InFlectis BioScience, France, during his PhD.

## Ethics Statement

Experiments were performed in accordance with the ethics committee in charge of animal experimentation of the Pays de la Loire, French law on animal welfare, EU Directive 2010/63/EU for animal experiments (#17858 and #32499), and the National Institutes of Health (NIH) Guide for the Care and Use of Laboratory Animals (NIH Pub. No. 85‐23, revised 2011).

## Conflicts of Interest

A.P. was supported by grants from InFlectis BioScience, France, during his PhD. The remaining authors declare no conflicts of interest.

## Supporting information




**Supporting File 1**: mco271056‐sup‐0001‐SuppMat.docx.

## Data Availability

The data that support the findings of this study are available from the corresponding author upon reasonable request.
