# Supplementary material for: O‐GlcNAcylation Stimulation Modulates Na+/K+ ATPase and Improves Outcomes in Hemorrhagic Shock
Source: MedComm (2020). 2026 Oct 11;7(10):e71056. doi: 10.1002/mco2.71056 (PMC13655371; doi:10.1002/mco2.71056)
Supplement: Supplementary file 1 [file MCO2-7-e71056-s001.docx]

**SUPPLEMENTARY INFORMATION**

**O-GlcNAcylation stimulation modulates Na+/K+ ATPase and improves outcomes in hemorrhagic shock**

Thomas Dupas^1^, Amandine Vergnaud^1^, Virginie Aillerie^1^, Martin Bouaud^1^, Angélique Erraud^1^, Thomas Pelé^1^, Antoine Persello^1^, Barbara Ribeiro de Oliveira^1^, Jérôme Montnach^1^, Aurore Girardeau^1^, Jules Lecomte^1^, Nolwenn Tessier^1^, Mathilde Guyon^1^, Angélique Blangy-Letheule^1^, Matthieu Rivière^2^, Jacques Lebreton^2^, Arnaud Tessier^2^, Edith Bigot-Corbel^3^, Aurélia A. Leroux^1,4^, Michel De Waard^1^, Nicolas J. Prat^5^, Bertrand Rozec^1^, Manon Denis^1^ and Benjamin Lauzier^1^

^1^ Nantes Université, CHU Nantes, CNRS, INSERM, l’institut du thorax, F-44000 Nantes, France

^2^ Nantes Université, CNRS, CEISAM, UMR 6230, 2 rue de la Houssinière, BP 92208, F-44000 Nantes, France

^3^ Nantes Université, CHU de Nantes, Departement of Biochemistry, F-44000 Nantes, France

^4^ Oniris, F-44300 Nantes, France

^5^ Institut de Recherche Biomédicale des Armées (IRBA), 91220 Bretigny-sur-Orge, France

**Corresponding authors:**

Thomas Dupas, l’institut du thorax, 8 quai Moncousu, 44000 Nantes, France.

E-mail: [thomas.dupas@univ-nantes.fr](mailto:thomas.dupas@univ-nantes.fr)

Benjamin Lauzier, l’institut du thorax, 8 quai Moncousu, 44000 Nantes, France.

E-mail: [benjamin.lauzier@univ-nantes.fr](mailto:benjamin.lauzier@univ-nantes.fr)

**Supplemental Figures**

**Figure S1. Bicarbonate levels and arterial pH in the different groups.** *Measure of (****A****) bicarbonate concentration and (****B****) arterial pH 24 h after the hemorrhagic shock. The sham group underwent surgery only. Hemorrhagic shock group underwent hemorrhagic shock protocol. NButGT group underwent hemorrhagic shock with dual injection of NButGT at 10 mg/kg. Statistical analyses were assessed by one-way ANOVA (****A****) or Kruskal-Wallis test (****B****). HS: hemorrhagic shock. **: p<0.01. n = 11-14.*

**Figure S2. Hemorrhagic shock and NButGT do not impact Na⁺/K⁺ ATPase expression in the heart.** *(****A-I****) Evaluation of Na^+^/K^+^ ATPase pump expression in the heart of (****A-C****) sham, (****D-F****) hemorrhagic shock or (****G-I****) NButGT treated rats 24 h after the hemorrhagic shock. Panels* ***C****,* ***F*** *and* ***I*** *represent merged images. Scale bar: 150 µm. (****J****) Evaluation by Western blot of Na^+^/K^+^ ATPase pump expression in the heart of sham, hemorrhagic shock or NButGT treated rats 24 h after the hemorrhagic shock (n=11-14). The sham group underwent surgery only. Hemorrhagic shock group underwent hemorrhagic shock protocol. NButGT group underwent hemorrhagic shock with dual injection of NButGT at 10 mg/kg. Statistical analyses were assessed by one-way ANOVA (****J****). HS: hemorrhagic shock.*

**Figure S3. NButGT efficiently increases O-GlcNAcylation levels in HEK 293 cells.** *Evaluation by Western blot of O-GlcNAcylation levels in HEK 293 cells. CTRL (control): untreated cells. NButGT: cells treated with NButGT at 10 µM for 24 h. n = 2.*

**Figure S4. NButGT does not affect cell viability in HEK 293 cells.** *Cell toxicity was evaluated by flow cytometry using propidium iodide staining. (****A****) A backgating strategy was used to analyze cells based on morphology (left panel), followed by doublet exclusion based on granularity (middle panel) and size (right panel). (****B****) Representative histogram of propidium iodide staining showing unstained cells (gray), staurosporine-treated cells (red) and untreated cells (orange). (****C****) Bar graph of percentage of propidium iodide-positive cells in untreated, NButGT-treated and staurosporine-treated cells. CTRL (control): untreated cells. NButGT: cells treated with NButGT at 0.1, 1, 10 or 100 µM for 24 h. Staurosporine: cells treated with staurosporine at 1 µM for 2 h. Staurosporine was used as positive control for cell toxicity. *: p<0.05 vs CTRL and NButGT 0.1, 1, 10, 100 µM groups. n = 3.*

**Figure S5. Timeline of hemorrhagic shock model.** *BGEM: blood gases electrolytes and metabolites. Created with BioRender.com.*

**Figure S6. Full unedited blots included in the figures.** *Quantified band of OGA is indicated by an asterisk*

**Supplemental Tables**

**Table S1. Antibodies used in Western blot experiments**

| **Target** | **Primary antibody** | | **Secondary antibody** | |
| --- | --- | --- | --- | --- |
|  | Antibody | Dilution | Antibody | Dilution |
| **O-GlcNAc** | HRP Anti-*O*-linked  N-Acetylglucosamine [RL2]  Abcam #ab201995 | 1/10 000^#^ | / | / |
| **GFAT1** | GFAT1 (D12F4)  Cell Signaling #5322 | 1/500* | Anti-rabbit IgG,  HRP-linked antibody  Cell Signaling #7074 | 1/10000* |
| **GFAT2** | Anti-GFPT2 [EPR19095]  Abcam #ab190966 | 1/2000* | Anti-rabbit IgG,  HRP-linked antibody  Cell Signaling #7074 | 1/10000* |
| **OGT** | OGT (D1D8Q)  Cell Signaling #24083 | 1/400* | Anti-rabbit IgG,  HRP-linked antibody  Cell Signaling #7074 | 1/10000* |
| **OGA** | Anti-MGEA/OGA  Abcam #ab105217 | 1/5000* | Anti-rabbit IgG,  HRP-linked antibody  Cell Signaling #7074 | 1/10000* |
| **Na^+^/K^+^ ATPase** | Anti-alpha 1 Sodium Potassium ATPase [464.6]  Abcam #ab7671 | 1/10000* | Anti-mouse IgG,  HRP-linked antibody  Cell Signaling #7076 | 1/10000* |
| **PAN-CADHERIN** | Pan-cadherin Polyclonal Antibody (Invitrogen #71-7100) | 1/30000* | Anti-rabbit IgG,  HRP-linked antibody  Cell Signaling #7074 | 1/10000* |
| **GAPDH** | GAPDH Antibody (6C5)  (Santa Cruz #sc-32233) | 1/5000* | Anti-mouse IgG,  HRP-linked antibody  Cell Signaling #7076 | 1/10000* |

*Dilutions carried out in 3% Bovine Serum Albumin (^#^) or in 5% milk (*).*

**Table S2: Wheat Germ Agglutinin gels composition**

| **Chemical product (µL)** | **Resolving gel** | **WGA gel** | **Stacking gel** |
| --- | --- | --- | --- |
| **WGA (10 mg/ml)** | / | 250 | / |
| **Acrylamide 37.5:1, 30%** | 2770 | 250 | 580 |
| **Tris/HCl 1.5M pH 8.8** | 1620 | 200 | / |
| **Tris/HCl 1M pH 6.8** | / | / | 1100 |
| **SDS** | 40 | 50 | 10 |
| **TEMED** | 4.67 | 1.5 | 4.67 |
| **Distilled water** | 2220 | 244 | 2710 |
| **APS 10% (after degassing)** | 22 | 4.5 | 22 |

*WGA: Wheat Germ Agglutinin; SDS: Sodium dodecyl sulfate; TEMED: N, N, N′, N′-Tetramethylethylenediamine, APS: Ammonium Persulfate*
